# Association of antiplatelet and anticoagulant use history with 30-day outcomes after lumbar puncture: a population-based retrospective study

**DOI:** 10.1007/s13760-026-03197-4

**Published:** 2026-09-27

**Authors:** Jinpyo Hong, David R. Hallan, Mason T. Stoltzfus, Kunal Koka, Manuel Bita, David Millar, Kyle Schantz, Andrea Shehaj, Naomi B. Gizaw, Elias B. Rizk

**Affiliations:** 1 Present Address: Penn State College of Medicine, Hershey, PA USA; 2 Department of Neurosurgery, Penn State Health Milton S. Hershey Medical Center, Hershey, PA USA; 3 Georgia State University, Atlanta, GA USA; 4 Department of Emergency Medicine, Massachusetts General Hospital, Boston, MA USA; 5 Department of Neurosurgery, Dell Medical School, The University of Texas at Austin, Austin, TX USA

**Keywords:** Lumbar puncture, Anticoagulant, Antiplatelet, Blood patch, Paralysis, Spinal decompression

## Abstract

**Objective:**

The safety of lumbar puncture in patients with a history of anticoagulant or antiplatelet use remains incompletely characterized. This study evaluated the coded occurrence of blood patch, paralysis, and spinal decompression within 30 days of lumbar puncture using a retrospective database.

**Methods:**

TriNetX, a database that houses clinical information from multiple healthcare entities, was used to carry out retrospective data collection and analysis. Data from patients with a history of antiplatelet use and anticoagulant use were collected separately. The timeline for outcomes of blood patch, paralysis, and spinal decompression started on the day of the first lumbar puncture and ended on the 30th day after the procedure. Propensity score matching was followed by statistical risk analyses.

**Results:**

A total of 13,310 patients were identified after propensity score matching for both lumbar puncture patients with antiplatelet use and lumbar puncture patients without antiplatelet, anticoagulant, heparin, or low-molecular-weight heparin use. Blood patch (OR [95% CI], 0.705 [0.563–0.882]) showed lower odds of association, while paralysis (OR [95% CI], 1.404 [1.097–1.798]) and spinal decompression (OR [95% CI], 2.225 [1.183–4.185]) showed greater odds of association; all were statistically significant. The corresponding absolute risk differences were 0.3% for paralysis and 0.1% for spinal decompression. A total of 25,788 patients were identified for both lumbar puncture patients with anticoagulant use and those without blood-thinner use after propensity score matching. Blood patch (OR [95% CI], 0.394 [0.332–0.467]) and spinal decompression (OR [95% CI], 0.659 [0.389–1.115]) showed lower odds of association, while paralysis (OR [95% CI], 1.154 [0.943–1.414]) showed greater odds of association; only blood patch was statistically significant.

**Conclusions:**

Among patients with a history of antiplatelet use, coded paralysis and spinal decompression within 30 days of lumbar puncture were associated with higher odds, although the absolute risk differences were small at 0.3% and 0.1%, respectively. Anticoagulant use history was not significantly associated with either paralysis or spinal decompression. Blood patch use was significantly lower in both exposure analyses. Since the TriNetX database cannot establish whether these coded outcomes were caused by lumbar puncture, these associations should be interpreted cautiously and require chart-level confirmation.

## Introduction

A lumbar puncture (LP), also commonly known as a spinal tap, is a procedure in which a needle is inserted into the lumbar region of the spinal column and advanced into the subarachnoid space. This is utilized to collect cerebrospinal fluid (CSF) for diagnostic or therapeutic purposes. There are various diagnostic purposes for LPs, such as evaluation of infectious diseases including meningitis or encephalitis [1]. Another diagnostic utilization is for specific neurological disorders such as multiple sclerosis through analysis for oligoclonal bands, or Guillain-Barre syndrome [2], LPs can also be utilized in the diagnosis of subarachnoid hemorrhage by investigating for xanthochromia. Regarding therapeutic purposes, LPs can be utilized for intrathecal drug administration and relieving increased intracranial pressure in certain conditions [3]. LPs are generally relatively safe procedures and rarely cause complications [4].

Anticoagulation and antiplatelet pharmacology are utilized for medical management of conditions associated with increased risk of thrombosis or embolism [5]. Vitamin K antagonists (warfarin), direct oral anticoagulants (dabigatran, rivaroxaban, apixaban, and edoxaban), and heparins (unfractionated heparin and low-molecular-weight heparin) are common anticoagulants. These agents are commonly utilized for conditions such as atrial fibrillation, deep vein thrombosis, or pulmonary embolism. Aspirin (acetylsalicylic acid), P2Y12 inhibitors (clopidogrel, prasugrel, and ticagrelor), and glycoprotein IIb/IIIa inhibitors (abciximab, eptifibatide, and tirofiban) are common types of antiplatelet agents. These agents are typically utilized for secondary prevention of myocardial infarction and stroke, acute coronary syndrome, post-percutaneous coronary intervention, and peripheral artery disease.

Despite their extensive indications for the above conditions, antiplatelets and anticoagulants can increase bleeding risk. LPs can cause spinal hematomas, which are collections of subdural or epidural hemorrhage in the space between the spinal arachnoid and dura and the space between the dura mater and vertebral surface, respectively. Spinal hematomas can cause progressive motor and sensory disturbances leading to spinal paralysis [6, 7]. Despite these outcomes, spinal hematomas are rare neurosurgical emergencies, but when they occur they can have significant outcomes [8, 9]. Furthermore, hematomas may exacerbate the need for spinal decompression or blood patches. Some literature has suggested a link between antiplatelets such as clopidogrel and the risk of spontaneous epidural hematomas [10]. However, use of these pharmacological agents in the context of lumbar punctures has not been well studied.

Therefore, the goal of this study was to evaluate associations between a history of antiplatelet or anticoagulant use and the coded occurrence of paralysis, spinal decompression, and blood patch within 30 days of LP. We hypothesized that medication exposure history would be associated with differences in these 30-day outcomes.

## Methods

### Study design

This retrospective cohort study used the TriNetX Research Network database, which includes data from multiple healthcare networks across the world, to query patients who underwent lumbar punctures with and without anticoagulant and antiplatelet use using the International Classification of Diseases, 10th Revision (ICD-10), Current Procedural Terminology (CPT) codes, RxNorm, and VA formulary medication systems [11]. TriNetX is a de-identified patient database that uses electronic medical records from multiple institutions worldwide. No Institutional Review Board (IRB) approval or patient consent was required due to the de-identified nature of the database. Previous literature confirmed the utility and validity of this database, and its details are well described [12–14].

The subdural and epidural hematoma outcomes from this broader study cohort have been published previously [11]. The present manuscript reports the additional outcomes of blood patch, paralysis, and spinal decompression.

### Patient selection

The TriNetX database was queried on September 25, 2022, for patients of any age and included outcomes from LPs with and without anticoagulant or antiplatelet use that occurred within the past 20 years. Patients who underwent lumbar punctures were selected using CPT codes 00635, 62,270, 1,035,653, and 62,328 and ICD-10 codes 009U3ZX, 009U3ZZ, 009U30Z, 009U4ZX, 009U40Z, and 009U4ZZ. A control cohort included any patients who underwent any of the aforementioned LP procedures and were not exposed to anticoagulants or antiplatelets. Patients in the anticoagulant-receiving cohort were further classified as having been exposed to enoxaparin, heparin, and other anticoagulants using RxNorm codes 67,108 and 5224, along with VA formulary code BL110, respectively. Patients in the antiplatelet cohort were selected using RxNorm codes 32,968, 10,594, 1191, 1,116,632, 3521, and 613,391 for exposure to clopidogrel, aspirin, prasugrel, and more. Patients who received protamine sulfate, idarucizumab, platelet transfusions, prothrombin, blood, or blood components were excluded due to the potential reversal of anticoagulation.

Using CPT codes, the resulting patient cohorts were examined for covariates that may confound results, including thrombocytopenia, von Willebrand disease, platelet defects, age, sex, demographics, and more. The cohorts were then propensity score-matched in an attempt to mimic true randomization.

### Statistical analysis

Various statistical tools, including risk difference (formerly referred to as attributable risk), odds ratio (OR), risk ratio (RR, also known as relative risk), and standard mean difference (SMD), were used to analyze data. Risk difference is helpful for quantifying the difference in risk of experiencing an outcome between exposed and unexposed cohorts. The risk ratio quantifies the ratio of the risk of experiencing an outcome between exposed and unexposed cohorts. The odds ratio quantifies the odds of experiencing an outcome between exposed and unexposed cohorts [15]. SMD quantifies the difference between cohorts, where a value of zero indicates no difference between cohorts [16]. An alpha level of 0.05 was used to determine statistical significance. Data were analyzed using TriNetX software with Java, R, and Python programming languages.

Outcomes were examined within 30 days of LP included paralysis, requirement of an epidural blood patch (EBP), and requirement of spinal decompression. The 30-day window was selected to focus the analysis on outcomes occurring in temporal proximity to LP; however, occurrence within this window does not establish that an outcome was caused by the LP.

## Results

Before propensity score matching, we identified 26,704 lumbar puncture patients with anticoagulant use history and 165,605 lumbar puncture patients without antiplatelet, anticoagulant, heparin, and LMWH (“blood thinner”) use. Age at index was 48.8 +/− 22.2 years and 28.5 +/− 23.2 years for anticoagulant-exposed and unexposed cohorts, respectively. A total of 47.8% of the anticoagulant-exposed cohort and 44.5% of the non-anticoagulant-exposed cohort were male. Pre-propensity score-matching baseline characteristics and demographics are shown in Table 1 for the anticoagulant versus non-anticoagulant comparative study.

**Table 1:** Pre-propensity score-matching baseline characteristics and demographics of lumbar puncture patients with and without anticoagulant use history

Baseline characteristic	Anticoagulant-exposed	% of Cohort	Non-anticoagulant-exposed	% of cohort	*p*-value	Standard mean difference
Patients	26,704	100%	165,605	100%		
Age at Index	48.8 (+/− 22.2 SD)	100%	28.5 (+/− 23.2 SD)	100%	< 0.001	0.894
Male	12,756	47.8%	66,381	44.5%	< 0.001	0.066
Female	13,905	52.2%	82,666	55.5%	< 0.001	0.066
White	16,756	62.8%	92,751	62.2%	0.053	0.013
Black or African American	5,356	20.1%	25,216	16.9%	< 0.001	0.082
Asian	567	2.1%	2,798	1.9%	0.006	0.018
Unknown Race	3,771	14.1%	27,230	18.3%	< 0.001	0.112
Fibrosis and Cirrhosis of Liver	533	2.0%	945	0.6%	< 0.001	0.120
Hypertensive Diseases	10,741	40.3%	20,179	13.5%	< 0.001	0.633
Diabetes Mellitus	4,958	18.6%	8,065	5.4%	< 0.001	0.414
Overweight, Obesity, Other Hyperalimentation	3,936	14.8%	10,279	6.9%	< 0.001	0.255
Nicotine Dependence	4,322	16.2%	11,080	7.4%	< 0.001	0.274
Alcohol Abuse	1,229	4.6%	2,131	1.4%	< 0.001	0.187
Alcohol Dependence	1,184	4.4%	1,745	1.2%	< 0.001	0.199
Chronic Lower Respiratory Diseases	4,145	15.5%	12,992	8.7%	< 0.001	0.210
Atrial Fibrillation and Atrial Flutter	1,810	6.8%	1,497	1.0%	< 0.001	0.302
Heart Failure	2,011	7.5%	2,129	1.4%	< 0.001	0.299
Ischemic Heart Diseases	3,463	13.0%	3,176	2.1%	< 0.001	0.420
Other Peripheral Vascular Diseases	906	3.4%	1,265	0.8%	< 0.001	0.178
Thrombocytopenia, Unspecified	1,906	7.1%	3,581	2.4%	< 0.001	0.224
Immune Thrombocytopenic Purpura	69	0.3%	186	0.1%	< 0.001	0.031
Qualitative Platelet Defects	50	0.2%	93	0.1%	< 0.001	0.035
Von Willebrand’s Disease	19	0.1%	97	0.1%	0.717	0.002
Hereditary Factor XI Deficiency	10	0.0%	12	0.0%	< 0.001	0.020
Disseminated Intravascular Coagulation	141	0.5%	271	0.2%	< 0.001	0.058
Hereditary Factor VIII Deficiency	10	0.0%	37	0.0%	0.243	0.007
Hereditary Factor IX Deficiency	10	0.0%	15	0.0%	0.001	0.018
Activated Protein C Resistance	107	0.4%	140	0.1%	< 0.001	0.062
Prothrombin Gene Mutation	87	0.3%	121	0.1%	< 0.001	0.054
Antiphospholipid Syndrome	107	0.4%	135	0.1%	< 0.001	0.063
Lupus Anticoagulant Syndrome	97	0.4%	131	0.1%	< 0.001	0.058

This table displays the baseline characteristics and demographics of lumbar puncture patients in the anticoagulant-exposed and unexposed cohorts before propensity score matching. An alpha level of 0.05 was selected for statistical significance

SD: standard deviation

We identified 14,395 antiplatelet-exposed lumbar puncture patients and 165,605 non-antiplatelet-exposed lumbar puncture patients before propensity score matching. Age at index was 59.2 +/− 17.8 years and 28.5 +/− 23.2 years for antiplatelet-exposed and unexposed cohorts, respectively. A total of 49.8% of the antiplatelet-exposed cohort and 44.5% of the non-antiplatelet-exposed cohort were male. Pre-propensity score-matching baseline characteristics and demographics are shown in Table 2 for the antiplatelet versus non-antiplatelet comparative study.

**Table 2:** Pre-propensity score-matching baseline characteristics and demographics of lumbar puncture patients with and without antiplatelet use history

Baseline characteristic	Antiplatelet-exposed	% of cohort	Non-antiplatelet-exposed	% of cohort	*p*-value	Standard mean difference
Patients	14,395	100%	165,605	100%		
Age at Index	59.2 (+/− 17.8 SD)	100%	28.5 (+/− 23.2 SD)	100%	< 0.001	1.485
Male	7,117	49.8%	66,381	44.5%	< 0.001	0.105
Female	7,176	50.2%	82,666	55.5%	< 0.001	0.105
White	9,413	65.9%	92,751	62.2%	< 0.001	0.076
Black or African American	2,758	19.3%	25,216	16.9%	< 0.001	0.062
Asian	234	1.6%	2,798	1.9%	0.042	0.018
Unknown Race	1,774	12.4%	27,230	18.3%	< 0.001	0.163
Fibrosis and Cirrhosis of Liver	365	2.6%	945	0.6%	< 0.001	0.154
Hypertensive Diseases	8,048	56.3%	20,179	13.5%	< 0.001	1.004
Diabetes Mellitus	4,076	28.5%	8,065	5.4%	< 0.001	0.647
Overweight, Obesity, Other Hyperalimentation	2,630	18.4%	10,279	6.9%	< 0.001	0.351
Nicotine Dependence	2,323	16.3%	11,080	7.4%	< 0.001	0.276
Alcohol Abuse	680	4.8%	2,131	1.4%	< 0.001	0.193
Alcohol Dependence	624	4.4%	1,745	1.2%	< 0.001	0.196
Chronic Lower Respiratory Diseases	2,791	19.5%	12,992	8.7%	< 0.001	0.314
Atrial Fibrillation and Atrial Flutter	1,804	12.6%	1,497	1.0%	< 0.001	0.474
Heart Failure	2,075	14.5%	2,129	1.4%	< 0.001	0.498
Ischemic Heart Diseases	3,556	24.9%	3,176	2.1%	< 0.001	0.706
Other Peripheral Vascular Diseases	882	6.2%	1,265	0.8%	< 0.001	0.292
Thrombocytopenia, Unspecified	1,326	9.3%	3,581	2.4%	< 0.001	0.296
Immune Thrombocytopenic Purpura	47	0.3%	186	0.1%	< 0.001	0.043
Qualitative Platelet Defects	49	0.3%	93	0.1%	< 0.001	0.062
Von Willebrand’s Disease	10	0.1%	97	0.1%	0.827	0.002
Hereditary Factor XI Deficiency	10	0.1%	12	0.0%	< 0.001	0.031
Disseminated Intravascular Coagulation	100	0.7%	271	0.2%	< 0.001	0.078
Hereditary Factor VIII Deficiency	10	0.1%	37	0.0%	0.002	0.021
Hereditary Factor IX Deficiency	10	0.1%	15	0.0%	< 0.001	0.030
Activated Protein C Resistance	99	0.7%	140	0.1%	< 0.001	0.096
Prothrombin Gene Mutation	83	0.6%	121	0.1%	< 0.001	0.087
Antiphospholipid Syndrome	102	0.7%	135	0.1%	< 0.001	0.099
Lupus Anticoagulant Syndrome	95	0.7%	131	0.1%	< 0.001	0.094

This table displays the baseline characteristics and demographics of the patients in the antiplatelet-exposed and unexposed cohorts before propensity score matching. An alpha level of 0.05 was selected for statistical significance

SD: standard deviation

After propensity score matching, 25,788 patients were identified for both lumbar puncture patient cohorts with and without anticoagulant exposure. Age at index was 47.9 +/− 22.0 years and 48.2 +/− 22.1 years, respectively. A total of 47.3% and 47.4% were male in the anticoagulant-use and non-anticoagulant-use cohorts, respectively. Post-propensity score-matching baseline characteristics and demographics are shown in Table 3 for the anticoagulant versus non-anticoagulant comparative study.

**Table 3:** Post-propensity score-matching baseline characteristics and demographics of lumbar puncture patients with and without anticoagulant use history

Baseline characteristic	Anticoagulant-exposed	% of cohort	Non-anticoagulant-exposed	% of cohort	*p*-value	Standard mean difference
Patients	25,788	100%	25,788	100%		
Age at Index	47.9 (+/− 22.0 SD)	100%	48.2 (+/− 22.1 SD)	100%		
Male	12,209	47.3%	12,216	47.4%	0.951	0.001
Female	13,577	52.6%	13,567	52.6%	0.930	0.001
White	16,179	62.7%	16,165	62.7%	0.899	0.001
Black or African American	5,173	20.1%	5,444	21.1%	0.003	0.026
Asian	542	2.1%	545	2.1%	0.927	0.001
Unknown Race	3,691	14.3%	3,441	13.3%	0.001	0.028
Fibrosis and Cirrhosis of Liver	493	1.9%	443	1.7%	0.099	0.015
Hypertensive Diseases	9,897	38.4%	9,945	38.6%	0.664	0.004
Diabetes Mellitus	4,432	17.2%	4,420	17.1%	0.889	0.001
Overweight, Obesity, Other Hyperalimentation	3,562	13.8%	3,473	13.5%	0.254	0.010
Nicotine Dependence	4,007	15.5%	4,096	15.9%	0.282	0.009
Alcohol Abuse	1,105	4.3%	1,094	4.2%	0.811	0.002
Alcohol Dependence	1,046	4.1%	966	3.7%	0.069	0.016
Chronic Lower Respiratory Diseases	3,772	14.6%	3,653	14.2%	0.136	0.013
Atrial Fibrillation and Atrial Flutter	1,364	5.3%	1,196	4.6%	0.001	0.030
Heart Failure	1,630	6.3%	1,442	5.6%	< 0.001	0.031
Ischemic Heart Diseases	2,766	10.7%	2,518	9.8%	< 0.001	0.032
Other Peripheral Vascular Diseases	739	2.9%	632	2.5%	0.003	0.026
Thrombocytopenia, Unspecified	1,650	6.4%	1,625	6.3%	0.652	0.004
Immune Thrombocytopenic Purpura	59	0.2%	51	0.2%	0.445	0.007
Qualitative Platelet Defects	40	0.2%	32	0.1%	0.345	0.008
Von Willebrand’s Disease	19	0.1%	19	0.1%	1.00	< 0.001
Hereditary Factor XI Deficiency	10	0.0%	10	0.0%	‘1.00	< 0.001
Disseminated Intravascular Coagulation	123	0.5%	110	0.4%	0.393	0.008
Hereditary Factor VIII Deficiency	10	0.0%	10	0.0%	‘1.00	< 0.001
Hereditary Factor IX Deficiency	10	0.0%	10	0.0%	‘1.00	< 0.001
Activated Protein C Resistance	89	0.3%	79	0.3%	0.440	0.007
Prothrombin Gene Mutation	74	0.3%	65	0.3%	0.445	0.007
Antiphospholipid Syndrome	90	0.3%	75	0.3%	0.242	0.010
Lupus Anticoagulant Syndrome	81	0.3%	72	0.3%	0.466	0.006

This table displays the baseline characteristics and demographics of the patients in the anticoagulant-exposed and unexposed cohorts after propensity score matching. An alpha level of 0.05 was selected for statistical significance

SD: standard deviation

We identified 13,310 lumbar puncture patients for both antiplatelet-exposed and non-antiplatelet-exposed cohorts after propensity score matching; age at index was 58.0 +/− 17.8 years and 59.0 +/− 18.0 years, respectively. A total of 49.0% of the antiplatelet-exposed cohort and 48.5% of the non-antiplatelet-exposed cohort were male. Post-propensity score-matching baseline characteristics and demographics are shown in Table 4 for the antiplatelet versus non-antiplatelet comparative study.

**Table 4:** Post-propensity score-matching baseline characteristics and demographics of lumbar puncture patients with and without antiplatelet use history

Baseline characteristic	Antiplatelet-exposed	% of cohort	Non-antiplatelet-exposed	% of cohort	*p*-value	Standard mean difference
Patients	13,310	100%	13,310	100%		
Age at Index	58.0 (+/− 17.8 SD)	100%	59.0 (+/− 18.0 SD)	100%	< 0.001	0.056
Male	6,520	49.0%	6,450	48.5%	0.391	0.011
Female	6,789	51.0%	6,855	51.5%	0.418	0.01
White	8,721	65.5%	8,631	64.8%	0.247	0.014
Black or African American	2,577	19.4%	2,689	20.2%	0.085	0.021
Asian	226	1.7%	268	2.0%	0.056	0.023
Unknown Race	1,679	12.6%	1,624	12.2%	0.307	0.013
Fibrosis and Cirrhosis of Liver	320	2.4%	305	2.3%	0.544	0.007
Hypertensive Diseases	7,088	53.3%	7,137	53.6%	0.547	0.007
Diabetes Mellitus	3,449	25.9%	3,396	25.5%	0.457	0.009
Overweight, Obesity, Other Hyperalimentation	2,225	16.7%	2,045	15.4%	0.003	0.037
Nicotine Dependence	2,100	15.8%	2,004	15.1%	0.103	0.020
Alcohol Abuse	614	4.6%	583	4.4%	0.359	0.011
Alcohol Dependence	543	4.1%	543	4.1%	1.00	< 0.001
Chronic Lower Respiratory Diseases	2,385	17.9%	2,249	16.9%	0.028	0.027
Atrial Fibrillation and Atrial Flutter	1,256	9.4%	1,065	8.0%	< 0.001	0.051
Heart Failure	1,510	11.3%	1,315	9.9%	< 0.001	0.048
Ischemic Heart Diseases	2,685	20.2%	2,333	17.5%	< 0.001	0.068
Other Peripheral Vascular Diseases	652	4.9%	561	4.2%	0.007	0.033
Thrombocytopenia, Unspecified	1,072	8.1%	988	7.4%	0.054	0.024
Immune Thrombocytopenic Purpura	39	0.3%	38	0.3%	0.909	0.001
Qualitative Platelet Defects	33	0.2%	25	0.2%	0.293	0.013
Von Willebrand’s Disease	10	0.1%	10	0.1%	1.00	< 0.001
Hereditary Factor XI Deficiency	10	0.1%	0	0%	0.002	0.039
Disseminated Intravascular Coagulation	81	0.6%	68	0.5%	0.286	0.013
Hereditary Factor VIII Deficiency	10	0.1%	10	0.1%	1.00	< 0.001
Hereditary Factor IX Deficiency	10	0.1%	10	0.1%	1.00	< 0.001
Activated Protein C Resistance	70	0.5%	58	0.4%	0.288	0.013
Prothrombin Gene Mutation	60	0.5%	49	0.4%	0.291	0.013
Antiphospholipid Syndrome	73	0.5%	59	0.4%	0.222	0.015
Lupus Anticoagulant Syndrome	66	0.5%	53	0.4%	0.232	0.015

This table displays the baseline characteristics and demographics of the patients in the antiplatelet-exposed and unexposed cohorts after propensity score matching. An alpha level of 0.05 was selected for statistical significance

SD: standard deviation

A secondary analysis of 25,788 patients was used to compare those who underwent lumbar puncture with anticoagulant use versus those without blood-thinner use. A different pattern of results was observed, with use of a blood patch indicating a significantly lower association (OR [95% CI], 0.394 [0.332–0.467]). Spinal decompression (OR [95% CI], 0.659 [0.389–1.115]) showed a non-significant trend toward lower odds. Paralysis was associated with higher odds (OR [95% CI], 1.154 [0.943–1.414]), but this association was not statistically significant (Table 5).

**Table 5:** Risk differences, risk ratios, and odds ratios for the coded occurrence of blood patch, paralysis, and spinal decompression within 30 days after lumbar puncture for patients with and without anticoagulant use history

Outcome	Risk difference	95% CI	*p*-value	Risk ratio	95% CI	Odds ratio	95% CI
Blood Patch	− 0.011	(− 0.013, − 0.009)	0.000	0.398	(0.337, 0.471)	**0.394**	**(0.332**,** 0.467)**
Paralysis	0.001	(− 0.000, 0.003)	0.165	1.153	(0.943, 1.410)	**1.154**	**(0.943**,** 1.414)**
Spinal Decompression	− 0.000	(− 0.001, 0.000)	0.118	0.659	(0.390, 1.115)	**0.659**	**(0.389**,** 1.115)**

This table displays the risk differences, risk ratios, and odds ratios comparing various outcomes experienced within 30 days of lumbar puncture by the anticoagulant exposed and unexposed cohorts. An alpha level of 0.05 was selected for statistical significance

CI: confidence interval

The bold is for all odds ratio values since these are the results of our study

**Fig. 1:**
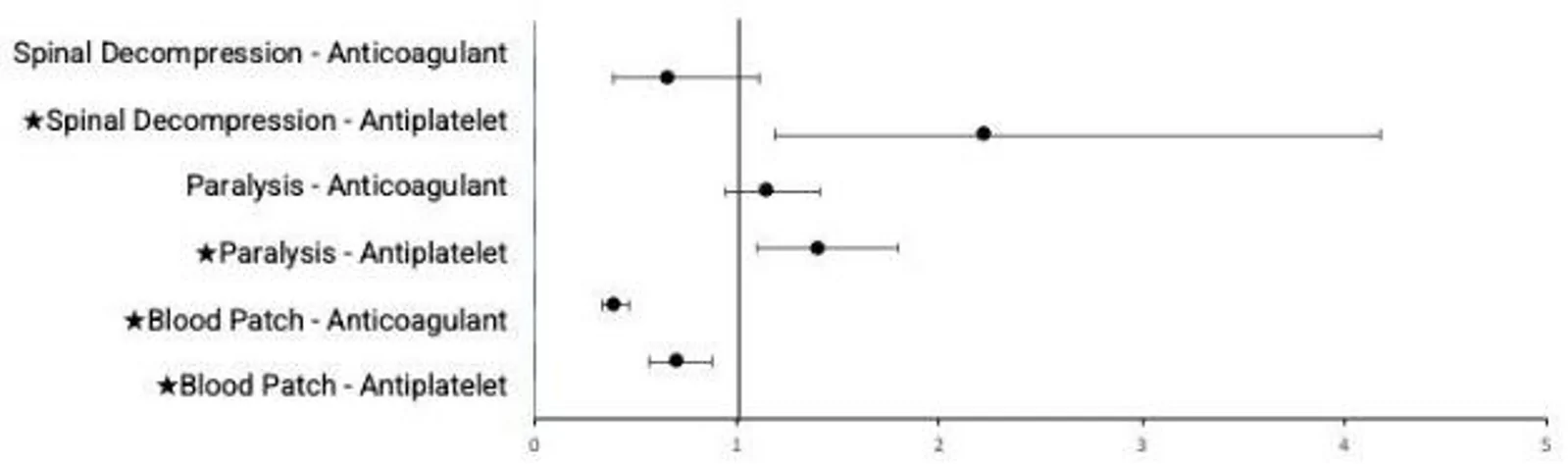
Forest plot of associations with 30-day outcomes in patients with and without anticoagulant and antiplatelet use history. This figure displays the odds ratios and 95% confidence intervals for blood patch, paralysis, and spinal decompression in patients with and without anticoagulant and antiplatelet use history. The star symbol denotes statistical significance

We conducted an analysis of 13,310 patients who underwent lumbar puncture and were matched based on antiplatelet use compared with no use of antiplatelets, anticoagulants, heparin, or low-molecular-weight heparin (LMWH). The analysis identified statistically significant differences in the coded occurrence of blood patch, paralysis, and spinal decompression within 30 days of LP. Use of a blood patch was associated with significantly lower odds, with an odds ratio (OR) of 0.705 and a 95% confidence interval (95% CI) of 0.563–0.882. Paralysis and spinal decompression were associated with significantly higher odds, with paralysis producing an OR [95% CI] of 1.404 [1.097–1.798] and spinal decompression an OR [95% CI] of 2.225 [1.183–4.185] (Table 6). These findings are summarized in Fig. 1.

**Table 6:** Risk differences, risk ratios, and odds ratios for the coded occurrence of blood patch, paralysis, and spinal decompression within 30 days after lumbar puncture for patients with and without antiplatelet use history

Outcome	Risk difference	95% CI	*p*-value	Risk ratio	95% CI	Odds ratio	95% CI
Blood Patch	− 0.004	(− 0.007, − 0.001)	0.002	0.708	(0.567, 0.884)	**0.705**	**(0.563**,** 0.882)**
Paralysis	0.003	(0.001, 0.006)	0.007	1.399	(1.096, 1.788)	**1.404**	**(1.097**,** 1.798)**
Spinal Decompression	0.001	(0.000, 0.002)	0.011	2.222	(1.183, 4.175)	**2.225**	**(1.183**,** 4.185)**

This table displays the risk differences, risk ratios, and odds ratios comparing various outcomes experienced within 30 days of lumbar puncture by the antiplatelet exposed and unexposed cohorts. An alpha level of 0.05 was selected for statistical significance

CI: confidence interval

The bold is for all odds ratio values since these are the results of our study

## Discussion

### Need for blood patch

The adverse outcome of requiring a blood patch following a lumbar puncture (LP) was evaluated in patients using anticoagulant and antiplatelet medications. An epidural blood patch (EBP) is an invasive procedure that uses a variable volume of a patient’s blood injected into the epidural space to seal a dural puncture and stop leakage of cerebrospinal fluid, which may be used following lumbar puncture [17]. Needing a blood patch is a recognized complication indicative of CSF leak and subsequent post-dural puncture headache (PDPH), or a “spinal headache,” thus classifying it as an adverse outcome [18, 19].

Our analysis revealed that both anticoagulant and antiplatelet cohorts exhibited a lower odds ratio (OR) of needing a blood patch post-lumbar puncture compared with their respective control groups. Specifically, the OR for both the anticoagulant group and the antiplatelet group was below 1. These results indicate that patients on anticoagulant and antiplatelet medications had less likelihood of developing this adverse outcome relative to those not on these medications. These findings are counterintuitive, as one would typically expect higher complication rates in anticoagulant and antiplatelet users due to the increased risk of bleeding and hemorrhage associated with these medications [20]. Our results suggest a different trend, as these patients appeared to encounter fewer PDPHs following LP, which may reduce the need for a blood patch.

Current guidelines recommend medication-specific management of anticoagulant and antiplatelet therapy before LP because of potential bleeding complications [7]. However, recent literature indicates that these concerns may be less pronounced for some antiplatelet agents. One study observed patients undergoing LP while on dual antiplatelet therapy and found few complications following LP.^21^ Other work has also evaluated LP in patients treated with ADP receptor antagonists [22]. Thus, delaying critical diagnostic procedures like LP in patients on specific anticoagulant and antiplatelet therapeutic regimens can postpone the diagnosis of treatable neurological emergencies and may increase cardiovascular risk when medications are withheld. It is therefore important to investigate the risks and benefits of timely LP in patients on different anticoagulant and antiplatelet therapies.

It is important to consider the lack of data associated with this niche topic, as conclusions are often based on observations from spinal anesthesia literature [23]. Such circumstances make translating these findings into clinical relevance difficult, as outcomes following LP in anticoagulant and antiplatelet patients remain unpredictable and relatively unexplored. While research continues to emerge, caution should still be considered when performing LP in this subgroup of patients.

### Paralysis

In our study, patients with a history of antiplatelet use had significantly greater odds of a paralysis code being recorded within 30 days of lumbar puncture compared with patients without a blood thinner use (OR [95% CI], 1.404 [1.097–1.798]). However, the absolute risk difference was 0.003, corresponding to 0.3%. The present database analysis cannot determine whether these paralysis diagnoses represented new complications directly attributable to lumbar puncture or were related to other clinical processes. Accordingly, this finding should be interpreted as an association rather than evidence that antiplatelet therapy increases the risk of lumbar-puncture-related paralysis. In contrast, anticoagulant use history was not significantly associated with coded paralysis within 30 days of LP (OR [95% CI], 1.154 [0.943–1.414]).

While generally safe, lumbar puncture procedures are associated with a range of common minor complications such as back pain, headaches, and puncture-site bleeding [24]. However, rare but severe complications can occur, including damaged nerves, infections, and spinal hematomas. [24]. Spinal hematomas have been associated with further serious complications, one of which is paralysis [24–26]. Paralysis following lumbar punctures can result from hemorrhage in the subdural or epidural spaces during the procedure [26]. This type of bleeding can lead to formation of a spinal hematoma, which can cause increased pressure on the spinal cord and make it vulnerable to neurological deficits, including paralysis [26, 27].

Several studies have explored the incidence and risk factors of different types of paralysis following lumbar puncture. For instance, Thömke et al. analyzed diagnostic lumbar puncture patient reports over a period of 14.5 years and found 2 patients who developed abducens palsy following lumbar puncture, yielding an incidence of approximately 1 abducens palsy in 5,800 diagnostic lumbar puncture cases [28]. Furthermore, post-lumbar-puncture paralysis may also develop as a result of post-procedure infection, as reported by da Silva and de Souza Loduca in a patient who developed meningitis symptoms following lumbar puncture and later developed lower-limb paralysis [29].

The observed association may warrant further investigation regarding the role of antiplatelet use history in risk assessment surrounding lumbar puncture. Alternative diagnostic approaches have been evaluated in specific clinical settings; for example, CT angiography has been studied as part of diagnostic strategies for aneurysmal subarachnoid hemorrhage that may avoid systematic use of lumbar puncture in selected patients [30]. However, because the present study cannot establish that the coded paralysis outcomes were caused by lumbar puncture, these findings should not by themselves be used to recommend alternative diagnostic strategies or changes in patient counseling based on antiplatelet use history. Chart-level confirmation is necessary before drawing such clinical conclusions.

### Need for spinal decompression

There was no statistically significant association between anticoagulant use history and coded spinal decompression within 30 days of lumbar puncture (OR [95% CI], 0.659 [0.389–1.115]). Spinal decompression is rarely performed following lumbar puncture and may be required in patients who develop a spinal hematoma or hemorrhage. A large retrospective cohort study found that in patients who were anticoagulated after lumbar puncture, only 5 of 342 patients needed spinal decompression after LP due to hematoma formation, even though 75 patients receiving anticoagulation developed hematomas [20]. While this study included patients who were anticoagulated after their LP, it highlights the rarity of spinal decompression following LP in patients with anticoagulation. Case reports have also described patients requiring spinal decompression due to hematoma or hemorrhage following LP [31]. Therefore, while spinal decompression following lumbar puncture may be of clinical significance in certain cases, statistical significance was not present for patients with anticoagulation in this study.

A history of antiplatelet use was associated with higher odds of a spinal decompression procedure code being recorded within 30 days of lumbar puncture (OR [95% CI], 2.225 [1.183–4.185]). However, the absolute risk difference was small at 0.1%. Spinal decompression may be required in the setting of spinal epidural or subdural hematoma. Case reports have described the need for spinal decompression in patients with a hematoma while receiving antiplatelet therapy [32]. However, the risk of spinal hematoma formation has been reported as minimal or absent in several studies of lumbar puncture or neuraxial procedures in patients receiving antiplatelet therapy [21, 33, 34]. These findings in the existing literature provide important clinical context but do not establish that the spinal decompression procedures identified in the present study resulted from lumbar-puncture related hematomas. Since TriNetX does not provide chart-level adjudication, imaging confirmation, or sufficient temporal detail to determine the indication for spinal decompression, the observed finding should be interpreted as an association between antiplatelet use history and coded spinal decompression within 30 days of lumbar puncture rather than evidence of a direct complication caused by the procedure. Given the small absolute risk difference, further chart-level investigation is needed to determine the clinical significance and etiology of this association.

### Limitations and prior publication

This study is limited by its retrospective database design, dependence on coded diagnoses and procedures, and the possibility of residual confounding after propensity score matching. Importantly, the 30-day outcome window establishes temporal proximity but does not establish causality. The database does not permit chart-level confirmation that each coded outcome was directly caused by the lumbar puncture. The subdural and epidural hematoma outcomes from the same broader study cohort were published previously [11]. The present manuscript is intended as a second analysis of the additional blood patch, paralysis, and spinal decompression outcomes, and this relationship should be disclosed transparently at submission. 

## Conclusions

In this retrospective database study, a history of antiplatelet use was associated with higher odds of coded paralysis and spinal decompression within 30 days of lumbar puncture, although the absolute risk differences were small at 0.3% and 0.1%, respectively. Anticoagulant use history was not significantly associated with either paralysis or spinal decompression. Blood patch use was significantly lower in both medication-exposure analyses. These findings demonstrate associations within a 30-day window and should not be interpreted as evidence that antiplatelet therapy causes neurological complications after lumbar puncture. Chart-level studies with clinical and imaging confirmation are needed to determine whether these coded outcomes were directly related to the lumbar puncture and to establish their clinical significance.

## Data Availability

The data that support the findings of this study are available from the corresponding author upon reasonable request.
